# Adjunctive intraoperative pulsed direct current with cefazolin: a rabbit proof-of-concept model for prevention of prosthetic joint infection

**DOI:** 10.3389/fcimb.2026.1927135

**Published:** 2026-09-22

**Authors:** Francisco Carmona-Torre, Martí Bernaus, José María Lamo-Espinosa, Andrés Valentí, Leire Fernández-Ciriza, Marta Rua, Ana Ramos, Gloria Abizanda, Diego Torres, Jose Antonio Calero, Lluis Font-Vizcarra, Jose L. del Pozo

**Affiliations:** 1 Infectious Diseases Division, Clínica Universidad de Navarra, Pamplona, Spain; 2 Biogipuzkoa Health Research Institute, Infectious Diseases Area, Infectious Diseases and Global Health Research Group, San Sebastián, Spain; 3 Department of Orthopedics and Traumatology, Osteoarticular Infection Unit, Hospital Universitari Mutua Terrassa, Terrassa, Spain; 4 Orthopedic and Traumatology Surgery Department, Hospital Biomédico ASCIRES, Valencia, Spain; 5 Institute for Research on Musculoskeletal Disorders, Catholic University of Valencia, Valencia, Spain; 6 Department of Orthopaedic Surgery and Traumatology, Clínica Universidad de Navarra, Pamplona, Spain; 7 Clinical Microbiology Department, Clínica Universidad de Navarra, Pamplona, Spain; 8 Diagnosis, Treatment and Prevention of Infectious Diseases Group, Navarra Institute for Health Research (IdiSNA), Pamplona, Spain; 9 Program of Regenerative Medicine, Centre for Applied Medical Research, University of Navarra, Pamplona, Spain; 10 Research and Development Department, AMES Powder Metallurgy Tech Center, Sant Vicent dels Horts, Spain; 11 Innovative Minds S.L., Terrassa, Spain; 12 Department of Orthopaedics and Traumatology, Osteoarticular Infection Unit, Hospital Moises Broggi, Consorci Sanitari Integral, University of Barcelona, Sant Joan Despí, Spain

**Keywords:** antibiotic prophylaxis, biofilms, *in vivo* model, prosthesis-related infections, pulsed direct current, *Staphylococcus aureus*

## Abstract

**Introduction:**

Perioperative bacterial contamination and rapid biofilm formation render prosthetic joint infection (PJI) a devastating complication in arthroplasty, where biofilms exhibit extreme resilience against conventional antibiotics. While low-amperage electrical stimulation can potentiate antibiotic activity *in vitro*, the prophylactic efficacy of high-amperage stimulation *in vivo* to prevent initial bacterial seeding remains untested.

**Methods:**

This study evaluated whether a brief, high-intensity pulsed direct current (pDC) applied during implantation, combined with systemic antibiotics, could prevent early colonization of implants and adjacent tissues. In a translational, proof-of-concept rabbit model, custom cobalt-chromium femoral implants were inoculated with *Staphylococcus aureus* ATCC 25923 and bilaterally inserted across four conditions: saline lavage (control); lavage + pDC (1 A, 2 Hz, 30 s); lavage + systemic cefazolin; or lavage + cefazolin + pDC. At 72 hours post-surgery, implants, peri-implant bone, and soft tissues underwent sonication and quantitative culture.

**Results:**

Saline lavage and pDC monotherapy failed to prevent heavy surface colonization and systemic dissemination, while cefazolin prophylaxis alone reduced bacterial bioburden but failed to achieve sterility. Remarkably, the combination of cefazolin and pDC achieved absolute clearance, rendering all implants, bone, and soft tissues culture-negative and preventing detectable systemic dissemination, with no clinical adverse events.

**Discussion:**

A single 30-second intraoperative pulse of high-amperage pDC, combined with standard cefazolin prophylaxis, completely prevented early periprosthetic infection by inhibiting initial bacterial attachment before mature biofilm assembly. These preliminary findings demonstrate a potential bioelectric-antibiotic synergy, providing a rapid, non-pharmacological, and workflow-compatible strategy to counter initial bacterial adhesion and the subsequent establishment of biofilms on orthopedic hardware.

## Graphical Abstract

**Figure:**
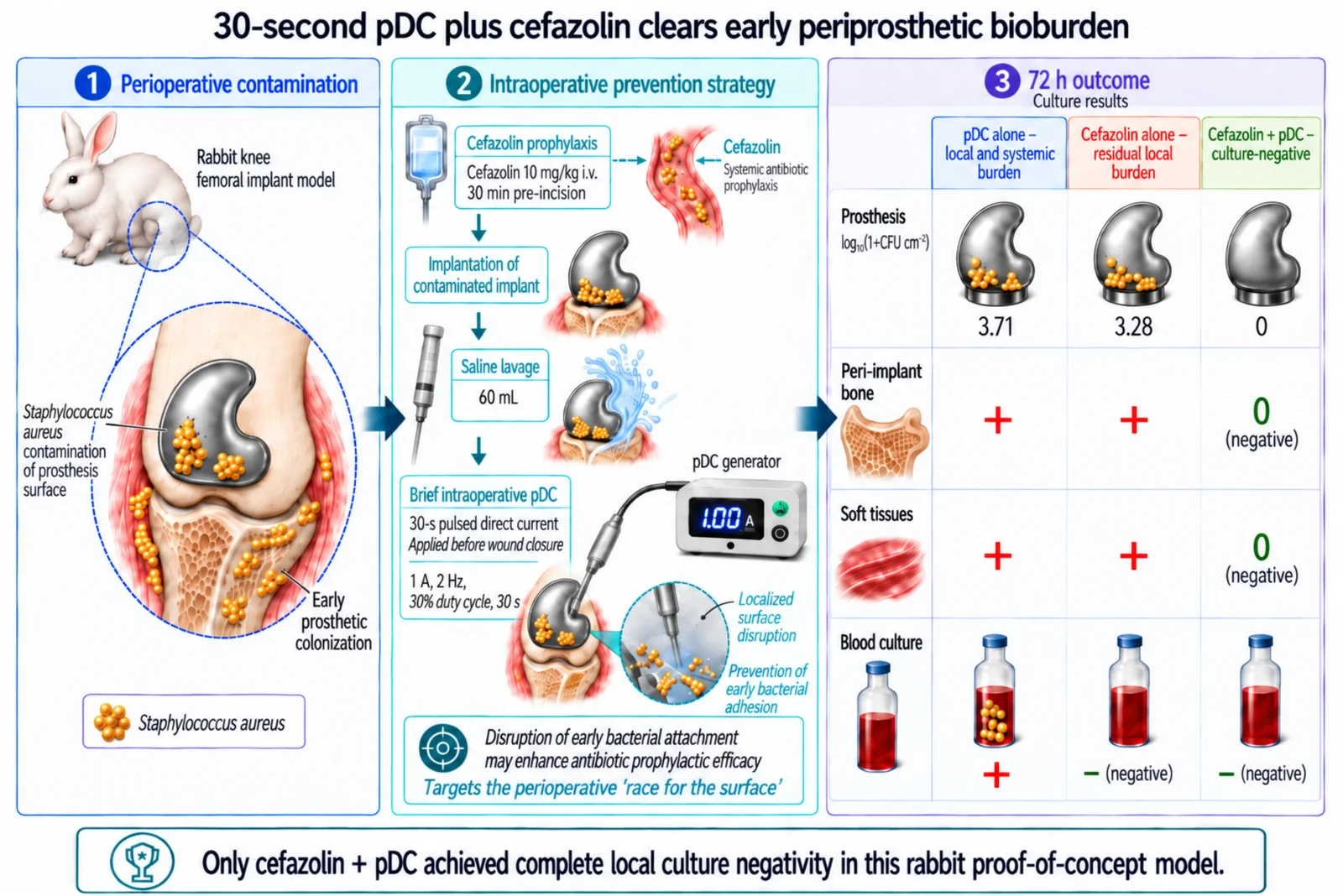

## 1 Introduction

Prosthetic joint infection (PJI) remains the most serious complication of arthroplasty, accounting for approximately 15% of revision procedures and contributing substantially to healthcare costs in the United States (Patel, 2023). Despite routine perioperative antibiotic prophylaxis, reported infection rates after primary hip and knee replacement range from 0.5% to 2%, escalating significantly in high-risk patients and trauma settings (Shoji and Chen, 2020; Visperas et al., 2022; Patel, 2023; MacConnell et al., 2024). Central to PJI pathogenesis is the rapid transition of free-floating bacteria into sessile surface-adherent communities. Once established, biofilms confer extreme resilience against both host immune responses and conventional antimicrobial therapies through reduced bacterial metabolic activity, restricted drug diffusion across the extracellular polymeric matrix, and localized microenvironmental shifts (Shoji and Chen, 2020; Visperas et al., 2022; MacConnell et al., 2024). In this regard, developing intraoperative strategies to disrupt initial bacterial adherence before recalcitrant biofilms can consolidate is of paramount clinical importance.

Low-level electrical stimulation (LLES) has emerged as a promising adjuvant approach against biofilm-mediated infection (Costerton et al., 1994; del Pozo et al., 2009; Visperas et al., 2022), modulating interfacial biophysical interactions to disrupt bacterial attachment and biofilm stability (Poortinga et al., 2002; van der Borden et al., 2005; Gall et al., 2013). Beyond these physical effects, LLES facilitates secondary processes, including electrically induced autolysis, reactive oxygen species (ROS) generation and localized shifts in pH and oxygen tension, that collectively enhance antibiotic penetration within the biofilm matrix (Zhang et al., 2014; Brinkman et al., 2016; Sen et al., 2020). Consequently, this synergy achieves significantly higher bacterial clearance than pharmacological treatment alone in *in vitro* studies (Shoji and Chen, 2020; Visperas et al., 2022; MacConnell et al., 2024).

In contrast, high-level electrical stimulation (HLES) achieves biofilm removal through a distinct, high-rate mechanism initiated by applying cathodic potentials beyond the hydrogen evolution reaction (HER) threshold. As demonstrated by Dargahi et al. (2014), polarization at potentials more negative than −1.5 V (vs. Ag/AgCl) triggers the vigorous electrolytic generation of hydrogen bubbles at the substrate interface. This ‘hydrogen pumping’ effect generates substantial interfacial shear force that mechanically displaces the extracellular polymeric matrix and embedded bacteria within seconds. This HLES-driven process can rapidly restore the underlying surface without compromising the substrate’s physicochemical integrity and provides a robust intervention when restricted antibiotic diffusion limits therapeutic efficacy, offering an exogenous bioelectric approach capable of neutralizing matrix-associated networks before mature protective assembly can occur.

However, a critical paradigm mismatch persists in current literature: the vast majority of electrical stimulation studies focus exclusively on eradicating established, mature biofilms in chronic infection models (Visperas et al., 2022; Shaikh et al., 2023). Consequently, the capacity of bioelectric phenomena—particularly HLES—to act as an immediate prophylactic barrier against initial bacterial seeding remains largely unexplored. While alternative anti-adherence strategies (such as novel biomaterials and antimicrobial coatings) are under active development, most face substantial translational and manufacturing hurdles (Shoji and Chen, 2020; Visperas et al., 2022; Ward et al., 2024). Furthermore, whereas limited LLES data suggest potential prophylactic utility (Del Pozo et al., 2014; Canty et al., 2017; Bernaus et al., 2022), virtually no evidence exists regarding the efficacy of HLES during the critical, transient window between initial bacterial attachment and early EPS matrix synthesis.

To address this critical knowledge gap, a reproducible rabbit arthroplasty model was established that recapitulates the immediate perioperative window. We hypothesized that brief, high-intensity pulsed direct current (pDC) delivered at the time of surgical implantation, applied as an adjunct to standard antibiotic prophylaxis, would prevent bacterial seeding of the prosthesis and peri-prosthetic tissues by disrupting initial bacterial attachment and countering early biofilm establishment. If validated, this rapid, workflow-compatible bioelectric intervention could be seamlessly integrated into routine arthroplasty protocols to prevent PJI at its point of origin.

## 2 Materials and methods

### 2.1 Bacterial strain and inoculum preparation

*Staphylococcus aureus* ATCC 25923, a well-characterized biofilm-forming reference strain, was selected as the model pathogen because it represents the primary high-virulence organism driving acute periprosthetic joint infections and exhibits rapid surface adherence and early matrix assembly (Diaz-Dilernia et al., 2026). This reference strain was sub-cultured twice on 5% sheep-blood agar (bioMérieux, Marcy-l’Étoile, France) at 37 °C for 24 hours. On the day of surgery, a bacterial suspension was prepared in 6 mL of brain–heart infusion (BHI) broth (Bacto™ Brain Heart Infusion, Becton Dickinson and Company, Le Pont de Claix, France). The suspension density was precisely adjusted to either a 0.3 or 0.5 McFarland standard (dependent on the specific experimental phase) using a calibrated turbidimeter (DensiCHEK plus, bioMérieux SA, Marcy-l’Etoile, France), corresponding to approximately 0.9 × 10^8^ CFU/mL and 1.5 × 10^8^ CFU/mL, respectively. Following a 15-min incubation at 37 °C to reactivate metabolic activity and initiate early-exponential growth, the custom implants were completely immersed in the bacterial inoculum for 30 s immediately prior to press-fit implantation. This immersion duration was validated in preliminary trials to ensure uniform, reproducible, high-density bacterial seeding across the prosthetic surface.

### 2.2 Custom implants and bioelectric stimulation setup

Custom-designed femoral knee prostheses were fabricated from medical-grade cobalt-chromium alloy (Co-Cr) bars (**Table 1**). The implants were anatomically engineered to replicate the distal femur configuration of the rabbit to ensure mechanical stability.

**Table 1:** Geometric specifications and exposed surface areas of the Co-Cr knee implant.

Element	Geometry	Exposed surface area
Articular cap	disc, Ø 4 mm × 1 mm height	0.2498 cm^2^
Intra-osseous stem*	cylinder, Ø 0.8 mm × 4 mm length	0.0528 cm^2^
**Total external area**	—	**0.3026 cm** **^2^**

*The proximal face of the stem is integral with the articular cap; only the lateral surface and distal face remain exposed.

Bold value indicates the calculated sum of the exposed surface areas of the articular cap and intra-osseous stem.

Implants were machined and steam-sterilized at 121 °C for 15 min using an Autoclave Autester ST dry PVL 25 L (J.P. Selecta S.A., Barcelona, Spain) prior to surgical insertion. All ancillary delivery components, including the needle electrodes used for current administration, were similarly steam-sterilized to maintain strict surgical sterility. The continuous, uninsulated metallic surface allowed uniform delivery of the electrical current across the joint interface.

The bioelectric stimulation system comprised a programmable external constant-current pulse generator. The pDC parameters were configured to deliver 1 A pulses at a frequency of 2 Hz with a 30% duty cycle for a total exposure time of 30 s. Building upon our previous *in vitro* proof-of-concept work utilizing constant-voltage pulse regimens (Bernaus et al., 2025), these constant-current parameters were optimized in preliminary benchtop trials to ensure reproducible cathodic hydrogen bubble generation and rapid interfacial bacterial detachment within a timeframe compatible with standard surgical workflows. The stimulator operated strictly in constant-current mode with a compliance voltage limit of 15 V.

### 2.3 Animal model and surgical procedure

Four male New Zealand White rabbits (weight range: 3.0–3.3 kg; sourced from Granja San Bernardo) were used in a bilateral, proof-of-concept experimental design, yielding a total of eight joint sites. Animals were housed individually under a controlled 12-h light/dark cycle with ad libitum access to standard laboratory chow and water.

To model intra-operative contamination of a joint arthroplasty, all surgical procedures were performed under strict aseptic conditions in a dedicated experimental operating suite using human-grade surgical preparation, sterile drapes, and standardized operating room protocols. General anesthesia was induced via intramuscular injection of ketamine (Imalgene 1000, Merial, Lyon, France) and xylazine (Rompun, Elanco, Greenfield, IN, USA), and maintained via inhalational isoflurane (Isovet, Piramal Critical Care, West Drayton, UK).

The total surgical duration was standardized across all procedures between 35 and 45 min. Following exposure of the distal femur, custom Co-Cr prostheses were contaminated by immersion in the bacterial suspension for 30 s as described above and press-fit implanted into the articular surface of the femoral condyles bilaterally within 15–20 min of bacterial exposure.

Immediately following implant placement, joint management was initiated: the articular space in control limbs received a 5-min sterile saline lavage (60 mL), whereas treatment groups received an identical 5-min saline irrigation combined with the intraoperative pDC pulse regimen delivered for 30 s. Following joint treatment, capsular and surgical wound closure were completed within 5–10 min.

### 2.4 Experimental stratification and study design

This exploratory pilot study followed an adaptive, sequential two-phase design. Each phase included two rabbits with bilateral implant placement, with one rabbit assigned to each treatment condition and both knees receiving the assigned treatment (**Table 2**). Phase 1 evaluated the standalone efficacy of the pDC monotherapy in the absence of systemic antibiotic prophylaxis under a high-density bacterial challenge (0.5 McFarland, ~1.5 × 10^8^ CFU/mL) to determine whether bioelectric stimulation alone could prevent local colonization. Based on Phase 1 outcomes demonstrating unmitigated colonization and systemic dissemination, Phase 2 was sequentially implemented to evaluate the translationally relevant strategy: combining intraoperative pDC with standard systemic cefazolin prophylaxis (10 mg kg^-1^ i.v.). In Phase 2, the bacterial inoculum was refined to 0.3 McFarland (~0.9 × 10^8^ CFU/mL) to establish a physiologically representative perioperative contamination level, enabling the evaluation of local surface adherence and early colonization under standard clinical prophylactic coverage over the 72-h study period (**Figure 1**).

**Table 2:** Experimental groups: systemic prophylaxis, bacterial inoculum, and intraoperative peri-closure interventions.

Phase	Cefazolin prophylaxis^a^	Inoculum (McFarland)	Limb treatment before closure	Rabbits (n)
1	–	0.5	60 mL saline lavage ± 30 s pDC^b^	1 control/1 pDC
2	10 mg kg^-1^	0.3	60 mL saline lavage ± 30 s pDC^b^	1 control/1 pDC

pDC, pulsed direct current. ^a^One dose 30 min pre-incision. ^b^1 A, 2 Hz, 30% duty cycle.

**Figure 1:**
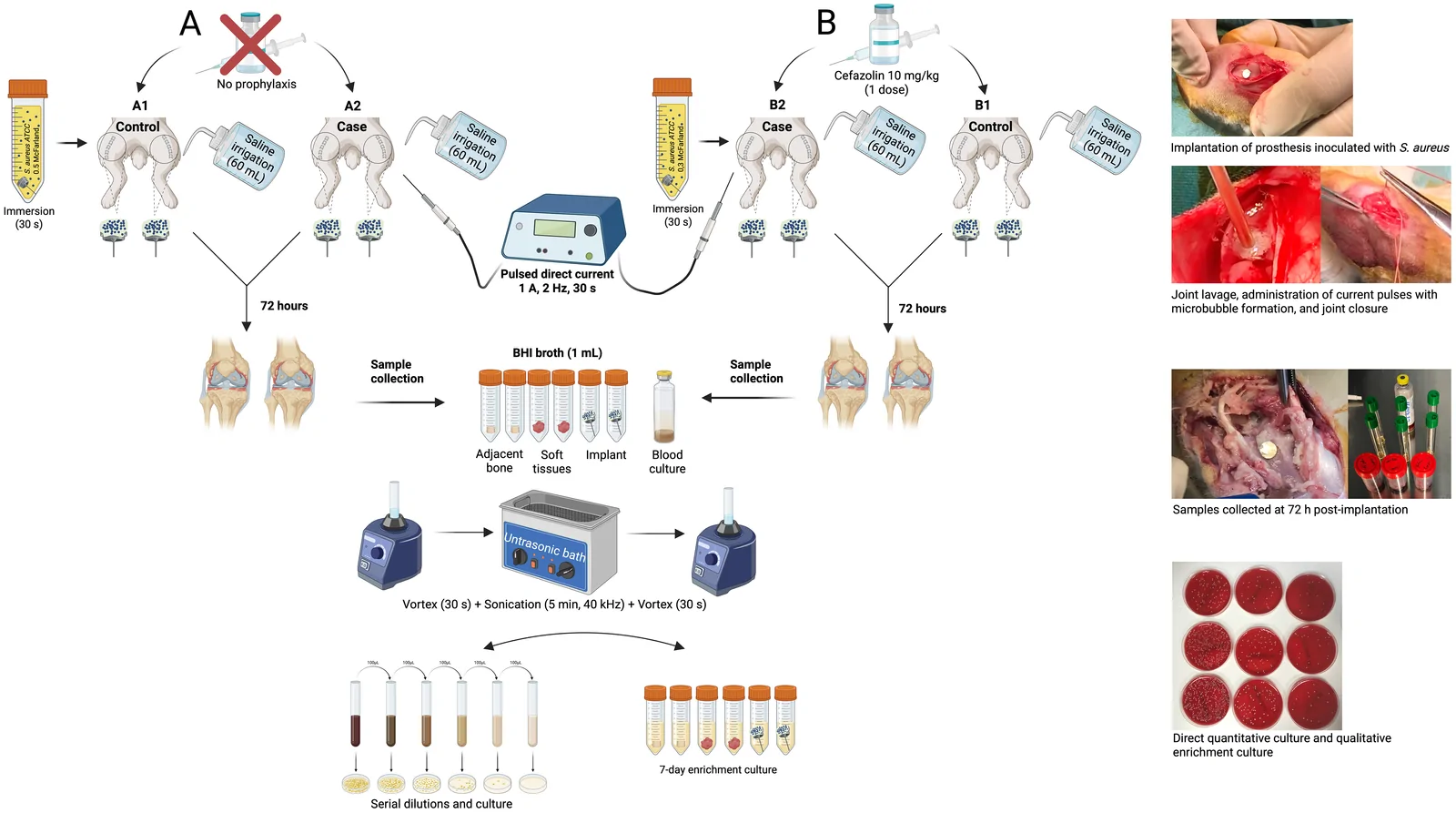
Experimental workflow for evaluating pulsed direct current (pDC) in a periprosthetic infection model. Study arms included **(A)** non-prophylaxis and **(B)** prophylactic cefazolin groups, comparing saline irrigation alone with saline irrigation plus pDC (1 A, 2 Hz, 30 s). Following intraoperative *S. aureus* inoculation and implantation, the prosthesis and periprosthetic tissues were harvested 72 hours post-surgery. Surface-adherent bacteria were recovered using a standardized vortex-sonication (5 min, 40 kHz) protocol, followed by quantitative serial dilutions and 7-day qualitative enrichment cultures. Right panels illustrate the surgical procedure, intraoperative pDC application with microbubble formation, and *ex vivo* sample processing. Created in BioRender. Carmona, F. (2026) https://BioRender.com/9px9749

### 2.5 Post-operative sampling and quantitative microbiological processing

At 72 h post-surgery, peripheral blood cultures were harvested immediately before the animals were humanely euthanized via an intravenous overdose of pentobarbital (200 mg kg^-1^ i.v.). The knee joints were aseptically dissected to retrieve the Co-Cr implant, adjacent cortical bone, and peri-articular soft tissues. Extra-articular organs (heart, liver, and both kidneys) were also aseptically harvested for systemic dissemination assessment.

To quantify sessile viable counts, explanted prostheses were immersed in 1 mL of BHI broth, vortexed for 30 s (Heidolph Instruments GmbH & Co. KG, Schwabach, Germany), sonicated for 5 min at 40 kHz and an acoustic power density of 0.4 W cm^-2^ in an Ultrasons-H water bath (J.P. Selecta, Barcelona, Spain), and vortexed again for an additional 30 s to maximize biofilm disruption.

This mechanical dislodgement workflow was adapted from established low-power bath sonication protocols for prosthetic-device microbiology (de Cueto et al., 2015), including the landmark 5-min protocol reported to preserve microbial viability (Trampuz et al., 2007). Furthermore, it is supported by *in vitro* evidence that Gram-positive organisms, including staphylococci, exhibit high resistance to ultrasound-associated viability loss under room-temperature conditions (Monsen et al., 2009; Silva et al., 2024).

In parallel, peri-implant bone and soft tissue specimens were processed in accordance with standardized tissue biopsy guidelines approved by the national clinical microbiology society (PNT-DBM-03) (de Cueto et al., 2015). Soft tissue samples were aseptically sectioned into small fragments on a sterile Petri dish using a sterile scalpel, whereas bone samples were processed whole owing to their limited dimensions. All biological samples were then immersed in 1 mL of BHI broth and subjected to the same ultrasonic disruption sequence to enhance bacterial yield from biological surfaces (Fang et al., 2021; Toyama et al., 2023).

Prosthesis, bone, and peri-implant soft tissue sonicates underwent 10-fold serial dilutions in BHI and were inoculated onto 5% sheep-blood agar plates for 24–48 h at 37 °C. For qualitative assessment of systemic dissemination, 100 µL of the undiluted BHI sonicate from each extra-articular organ specimen was plated directly onto 5% sheep-blood agar. To ensure detection of slow-growing or low-density residual colonization, the remaining volume of the BHI sonication broth was incubated at 37 °C as an enrichment culture for 7 days.

Bacterial bioburden was expressed as log_10_ CFU cm^-2^ for prosthetic surfaces and log_10_ CFU mL^-1^ for bone and tissue homogenates. The quantitative lower limit of detection (LOD) was defined as 10 CFU mL^-1^ for liquid matrices, corresponding to a mathematical limit of 1.52 log_10_ CFU cm^-2^ for the prosthesis surface area, defined by the growth of at least one colony from a 100 µL inoculum. Total bacterial eradication (reported as 0) was strictly defined as the complete absence of growth on both the primary agar plates and the 7-day enrichment broth. Phenotypic identity was verified by MALDI-TOF mass spectrometry (VITEK-MS, bioMérieux, Marcy-l’Étoile, France) and antibiotic susceptibility was monitored by automated microdilution (VITEK-2, bioMérieux, Marcy-l’Étoile, France).

### 2.6 Humane clinical endpoints

The study endpoints were predefined by the manifestation of severe systemic infection (sepsis) or a significant localized infection. Clinical indicators included fever >38.5 °C, spontaneous bleeding, or weight loss >20%, prompting immediate termination of the experiment.

Pain-related endpoints included lethargy, apathy, coat deterioration, teeth grinding, dehydration, muscle atrophy, reduced nocturnal fecal production, and decreased responsiveness to stimuli. If these signs persisted despite analgesic adjustment, euthanasia was performed following ethical guidelines.

### 2.7 Statistical analysis

Given the exploratory, proof-of-concept pilot nature of this study and the restricted cohort size, inferential hypothesis testing was deliberately omitted, and statistical analyses were confined to descriptive metrics. Consequently, data points are intended for hypothesis-generating purposes. Raw limb-level values are reported alongside descriptive summary metrics where appropriate.

Prosthetic bacterial burden was mathematically evaluated using the transformed scale log_10_(1+CFU cm^-2^) to accommodate zero counts systematically and mitigate right-skewness. To account for the variance in the initial bacterial challenge between experimental phases (No-prophylaxis vs. Prophylaxis), pDC treatment efficacy was determined as the within-stratum mean difference (Δmean) relative to its respective phase-matched saline lavage control. In this analytical framework, a Δmean > 0 denotes a net quantitative reduction in periprosthetic bioburden achieved by the bioelectric intervention.

## 3 Results

### 3.1 Phase 1: Local periprosthetic bioburden under non-prophylactic conditions

Phase 1 evaluated the baseline local contamination model and the potential antibacterial effect of intraoperative pulsed direct current (pDC) monotherapy in the absence of systemic antibiotic coverage. Animals were challenged with a high-density inoculum (0.5 McFarland, ~1.5 × 10^8^ CFU/mL) without perioperative antibiotic prophylaxis. Periprosthetic bacterial colonization (prosthesis, peri-implant bone, and soft tissue) was evaluated 72 hours post-inoculation.

In this non-prophylactic setting, persistent local contamination was observed across all animals regardless of the intraoperative local treatment applied. The animal receiving saline lavage alone (A1) exhibited high prosthetic bacterial loads in both knees (3.90 and 5.46 log_10_ CFU cm^-2^), accompanied by positive qualitative cultures in all periprosthetic bone and soft-tissue samples. In the animal receiving lavage combined with pDC (A2), prosthesis burden was reduced in one knee (1.52 log_10_ CFU cm^-2^) but remained high in the contralateral knee (5.89 log_10_ CFU cm^-2^). Bone and soft-tissue cultures for animal A2 similarly demonstrated persistent qualitative bacterial growth across both limbs (**Table 3**; **Figure 2**).

**Table 3:** Prosthesis, bone, and soft-tissue culture results 72 h after *S. aureus* inoculation.

Group	Animal	Treatment	Knee	Prosthesis^a^	Bone^b^	Soft Tissues^b^
No prophylaxis	A1	Lavage	R	3.90	+	+
No prophylaxis	A1	Lavage	L	5.46	+	+
No prophylaxis	A2	Lavage + pDC	R	1.52	+	+
No prophylaxis	A2	Lavage + pDC	L	5.89	+	+
Prophylaxis	B1	Lavage	R	3.36	2.7	2.72
Prophylaxis	B1	Lavage	L	3.20	3.26	3.87
Prophylaxis	B2	Lavage + pDC	R	0	0	0
Prophylaxis	B2	Lavage + pDC	L	0	0	0

^a^ log_10_(1+CFU cm^-2^); ^b^Bone and soft tissues expressed as log_10_(1+CFU mL^-1^) when quantitative values were available. pDC: pulsed direct current. R: Right; L: Left; “+”: qualitative growth, “0”: no detectable growth at the assay’s limit of detection.

**Figure 2:**
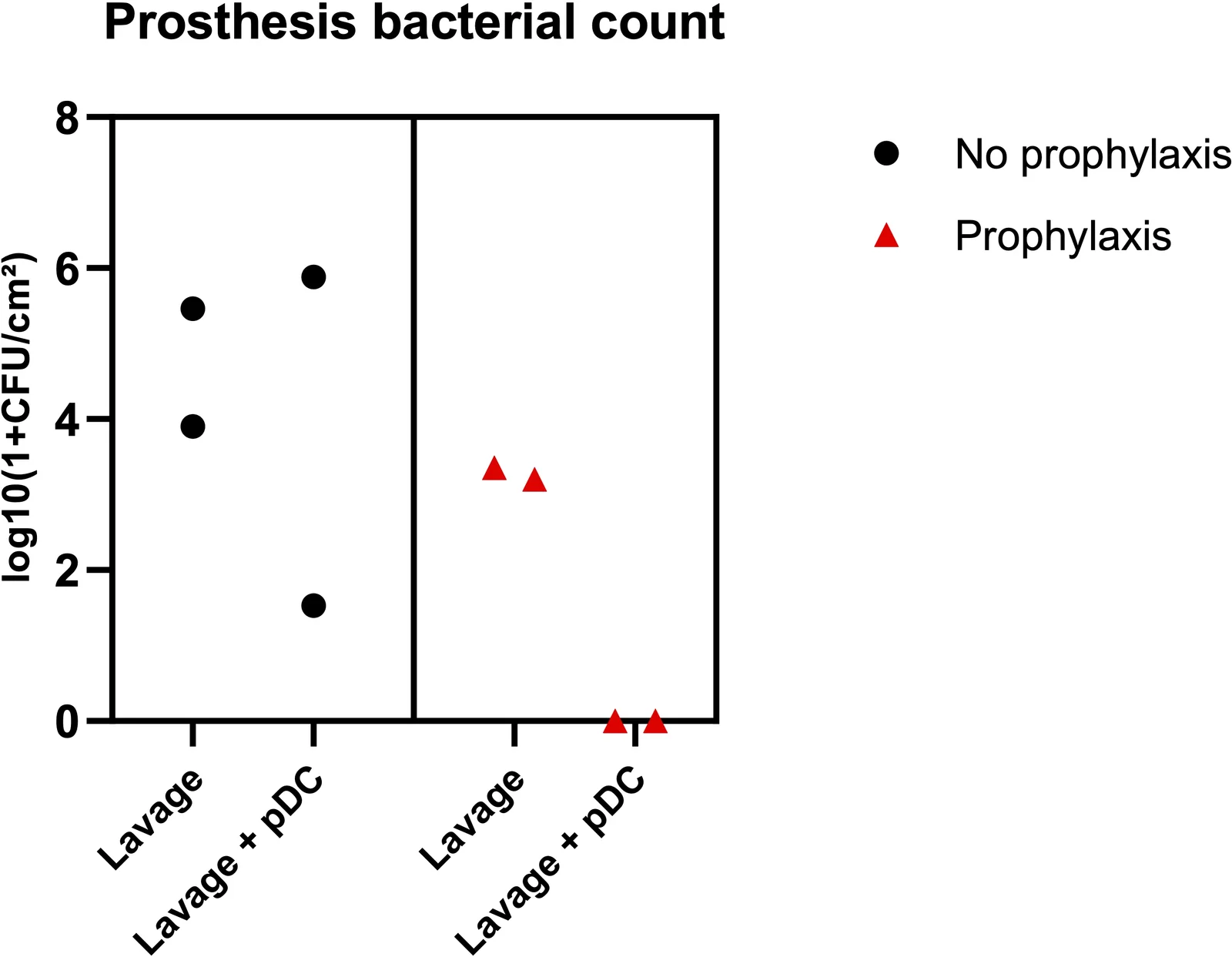
Efficacy of pulsed direct current (pDC) and antibiotic prophylaxis on prosthetic bacterial burden. *S. aureus* recovery from explanted prostheses 72 hours post-implantation. Data points represent individual samples expressed as log_10_(1+CFU cm^-2^). The combination of prophylactic cefazolin and pDC therapy (Lavage + pDC) achieved complete bacterial clearance across all specimens.

### 3.2 Phase 2: *In vivo* eradication efficacy of adjunctive pDC with systemic cefazolin prophylaxis

Phase 2 evaluated the eradication efficacy of combining intraoperative pDC with standard perioperative cefazolin prophylaxis (10 mg kg^-1^ i.v.) using a refined, clinically relevant inoculum (0.3 McFarland, ~0.9 × 10^8^ CFU/mL) to model intraoperative arthroplasty contamination. Periprosthetic bacterial colonization was evaluated at 72 hours post-inoculation.

Under systemic cefazolin prophylaxis, the control animal receiving saline lavage alone (B1) exhibited quantifiable prosthetic bacterial counts in both knees (3.36 and 3.20 log_10_ CFU cm^-2^), with persistent bacterial growth also detected and quantified in both peri-implant bone and soft tissues. By contrast, the animal treated with adjunctive pDC under cefazolin coverage (B2) demonstrated no detectable bacterial growth across all prosthetic, bone, and soft-tissue samples in both limbs (**Table 3**; **Figures 2**, **3**). This complete absence of local bacterial growth in animal B2 was confirmed by both negative primary agar plates and negative 7-day enriched BHI broth cultures.

**Figure 3:**
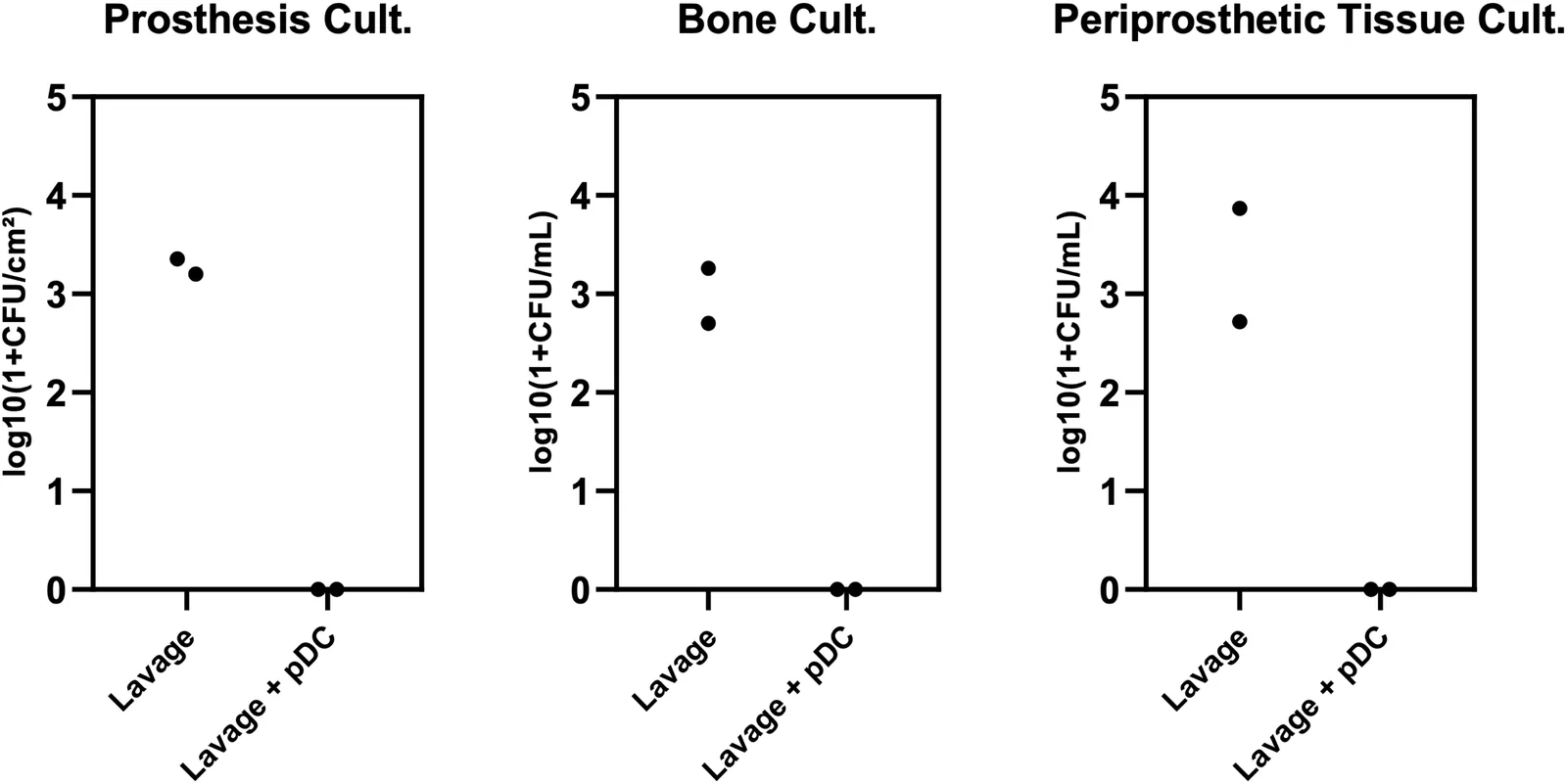
Adjuvant pulsed direct current (pDC) achieves total elimination of periprosthetic bacterial burden. Quantification of *S. aureus* burden in explanted prostheses, periprosthetic bone, and soft tissues 72 h post-implantation in animals receiving systemic cefazolin prophylaxis. Data points denote individual samples expressed as log10​(1+CFU cm^-2^) for prosthesis; log10(1+CFU mL^-1^) for bone and tissue. Adjuvant pDC therapy (Lavage + pDC) achieved complete bacterial clearance across all anatomical compartments compared to the prophylaxis-only control (Lavage).

### 3.3 Within-stratum efficacy of pDC effect

When expressed as within-stratum differences relative to the corresponding lavage control, the adjunctive pDC intervention was associated with a reduction in prosthesis bacterial burden in both strata (**Table 4**). This reduction was modest in the absence of systemic prophylaxis (Δmean = 0.97 log_10_ CFU cm^-2^). Under cefazolin prophylaxis, pDC was associated with no detectable prosthesis colonization (Δmean = 3.28 log_10_ CFU cm^-2^).

**Table 4:** Animal-level prosthesis burden and within-stratum Δmean log_10_(1+CFU cm^-2^) relative to the lavage control.

Group	Animal	Treatment	Prosthesis^a^	Δmean^b^
No prophylaxis	A1	Lavage	4.68	0
No prophylaxis	A2	Lavage + pDC	3.71	0.97
Prophylaxis	B1	Lavage	3.28	0
Prophylaxis	B2	Lavage + pDC	0	3.28

^a^ Animal-level mean of per-knee log10(1+CFU cm^-2^). ^b^Difference relative to the lavage-treated animal within the same prophylaxis stratum (positive values indicate reduction in prosthetic bacterial burden); pDC: pulsed direct current.

### 3.4 Extra-articular organ cultures and systemic dissemination

Systemic dissemination was assessed using qualitative blood cultures and qualitative cultures of extra-articular organs, including heart, liver, and a pooled bilateral kidney specimen. Consistent with this qualitative organ-culture workflow, organ results are reported as positive or negative. All recovered isolates were identified as *Staphylococcus aureus* by MALDI-TOF mass spectrometry.

In Phase 1, bacteremia was detected in both animals without systemic prophylaxis. In animal A1 (lavage), *S. aureus* was recovered from the liver and pooled bilateral kidney specimen, whereas the heart culture was negative. In animal A2 (lavage + pDC), *S. aureus* was recovered from the heart, liver, and pooled bilateral kidney specimen.

In Phase 2, blood cultures were negative in both animals receiving cefazolin prophylaxis. No bacterial growth was recovered from heart, liver, or pooled bilateral kidney cultures in either B1 or B2, either on primary plates or after 7-day enrichment.

### 3.5 Local tissue tolerability and implant integrity

Macroscopic post-mortem evaluation of the joint compartments at 72 h demonstrated excellent biological tolerability to the bioelectric regimen. No signs of tissue necrosis, severe inflammatory osteolysis, or thermal injury were observed in the peri-prosthetic bone or surrounding soft tissues following the 1 A intraoperative electrical surge. Additionally, the retrieved cobalt-chromium implants maintained full structural integrity with no signs of surface degradation, discoloration, or pitting corrosion under the constant-current stimulation profile.

## 4 Discussion

This proof-of-concept study demonstrates that the intraoperative application of high-amperage pDC, when synchronized with standard systemic cefazolin prophylaxis, achieves complete bacterial clearance of both the prosthetic surface and the surrounding osteoarticular tissues. Our data reveal a critical failure of current monotherapies: neither the “gold standard” of systemic antibiotics nor the high-intensity electrical intervention alone could prevent *Staphylococcus aureus* colonization. These results reinforce the paradigm that preventing PJI is not merely a task of reducing bacterial load, but of winning the “race for the surface” during the immediate perioperative window, before the assembly of a protective EPS matrix (Flemming et al., 2023; Turner et al., 2025).

To achieve rapid decontamination during the surgical procedure, current practice relies almost exclusively on intraoperative antiseptic irrigation, such as dilute povidone-iodine, chlorhexidine gluconate, or hydrogen peroxide washes, and local antibiotic deposition (e.g., intrawound vancomycin powder) (Zebala et al., 2014; Calkins et al., 2020). Although widely employed for their broad-spectrum activity, these local adjuvants fail to reliably eradicate bacteria already adhering to hardware surfaces (Ernest et al., 2018; Mulpur et al., 2024) and pose growing concerns regarding soft-tissue cytotoxicity, delayed wound healing, and impaired peri-implant osseointegration (Schmidt et al., 2018; Hamad et al., 2025). Alternative prophylactic strategies engineered for sustained action, including antibacterial coatings (e.g., silver or polypeptides), biomimetic antifouling topographies, and stimuli-responsive hydrogels, address a different clinical timeline; yet, their translation remains severely constrained by long-term biocompatibility issues, osteoblast toxicity, and the inevitable depletion of the antimicrobial reservoir (Ghimire and Song, 2021; Diez-Escudero et al., 2022; Liu et al., 2023; Semeshchenko et al., 2025). This leaves a critical therapeutic void for an intraoperative intervention that provides definitive, non-pharmacological bacterial clearance within the immediate perioperative window without compromising tissue integration or surgical workflow.

In this regard, electrical fields have emerged as a promising adjuvant for preventing or disrupting implant-associated biofilms. These technologies are generally categorized into alternating approaches, including alternating current (AC) fields, alternating magnetic fields (AMFs), and hybrid electromagnetic schemes, and direct-current (DC) modalities, such as continuous low-amperage DC, pulsed fields, and cathodic voltage-controlled electrical stimulation (CVCES). While AC and AMFs can disrupt early-stage adherent bacteria and established biofilms, they often suffer from a narrow therapeutic window due to collateral mammalian cell toxicity or limited *in vivo* efficacy (Bernaus et al., 2022; Beitl et al., 2025). In contrast, comparative studies show that DC is more effective than AC at detaching and killing bacteria on surgical stainless steel and titanium, up to 78% detachment with DC versus 31% with block AC under similar conditions (van der Borden et al., 2004). Importantly, several *in vivo* reports describe no adverse effects of DC on bone or surrounding soft tissues (Ehrensberger et al., 2015; Nodzo et al., 2016), although potential changes to the surface of titanium under DC have been noted and require careful consideration of dose and electrode design (Kaiser et al., 2020). Building on this, our study utilizes a 1 A high-amperage prophylactic pDC to achieve definitive surface debridement in the early stages of colonization, effectively preempting the development of a recalcitrant biofilm architecture.

The synergistic enhancement of antibiotic activity by low-intensity currents arises from a convergence of physicochemical and biological processes, including promoted bacterial autolysis, ROS-mediated membrane damage, electrolytic oxygenation, and electrophoretic drug delivery (Stoodley et al., 1997; Stewart et al., 1999; Zhang et al., 2014; Brinkman et al., 2016; Sen et al., 2020). While these mechanisms impair early bacterial colonization and facilitate matrix penetration through pH-induced shifts and the electrochemical generation of biocides (Stoodley et al., 1997; Del Pozo et al., 2014; Dusane et al., 2019; Czerwinska-Glowka and Krukiewicz, 2020), their translational utility is fundamentally constrained by the necessity for prolonged exposure (Canty et al., 2019). Existing literature consistently demonstrates that while low-amperage regimens significantly reduce bacterial adhesion across various models, absolute sterility is rarely achieved without multi-day treatments, a timeline often incompatible with acute surgical interventions (Del Pozo et al., 2009; Del Pozo et al., 2014; Voegele et al., 2015; Schmidt-Malan et al., 2017; Munoz-Mahamud et al., 2018; Tamimi et al., 2024).

By contrast, HLES overcomes these temporal constraints by shifting the therapeutic paradigm toward rapid, biophysically driven surface clearance. By driving cathodic potentials beyond the hydrogen evolution reaction (HER) threshold, HLES triggers vigorous interfacial gasification (Dargahi et al., 2014; Schneider et al., 2018), inducing a ‘hydrogen pumping’ effect, that generates interfacial shear forces capable of physically detaching early-stage adherent bacteria as well as decoupling any nascent extracellular polymeric matrix and its embedded pathogens from the substrate within seconds (Dargahi et al., 2014). This rapid mechanical cleansing is synergistically complemented by concomitant localized alkalization (pH > 12) that compromises bacterial viability through membrane depolarization, ATP synthesis inhibition and the induction of catastrophic oxidative stress (Canty et al., 2017; Tan et al., 2018). Crucially, intense local gasification and the resulting abrupt, highly alkaline microenvironment may collapse the nascent bioelectrical niche that supports early surface colonization, disrupting extracellular electron transfer–mediated coordination and preventing microcolony consolidation into a resilient, matrix-protected biofilm (Nostro et al., 2012; Kato, 2015; Schilcher and Horswill, 2020).

The 1 A pDC employed in this study represents a paradigmatic technical shift from the low-intensity regimens (typically <10 mA) that have historically defined bioelectric research. By prioritizing rapid ablation over gradual enhancement and targeting pathogens in their planktonic or nascent states at the point of implantation, this protocol exploits a critical biological window. Ultimately, this approach preempts recalcitrant biofilm assembly while aligning therapeutic intensity with the practical constraints of the surgical workflow (Ehrensberger et al., 2015; Nodzo et al., 2015; Nodzo et al., 2016; Schneider et al., 2018; Weeks et al., 2023; Zhu et al., 2024).

A pivotal observation in this study is the divergence between the absolute sterilization reported in idealized *in vitro* HLES models and our current *in vivo* observations. While Schneider et al. (2018) achieved total biofilm clearance using a lower current (300 mA) in aqueous environments, 1 A alone failed to achieve periprosthetic clearance in our animal model in the absence of cefazolin. This disparity underscores the limitations inherent to idealized benchtop models, where optimized electrolytes and the absence of anatomical constraints facilitate uniform current distribution and unobstructed gas expansion. Under these idealized conditions, once adherent bacteria or nascent matrices are mechanically displaced from a smooth surface, they are effectively sequestered from the substrate, a procedural artifact that precludes the potential for immediate re-attachment within the experimental window. However, as demonstrated by Toma et al. (2018), surface decontamination *in vitro* does not inherently prevent rapid recolonization, particularly when detached bacteria or cellular aggregates remain within the immediate periprosthetic microenvironment.

Beyond the spatial constraints of the surgical site, the temporal dynamics of microbial assessment also play a decisive role. While *in vitro* efficacy is often measured immediately post-intervention, our *in vivo* model incorporates a 72-hour delay before necropsy. This window is biologically critical, as it allows for the potential proliferation of residual microorganisms or hematogenous reseeding from adjacent tissue reservoirs (Urish et al., 2016; Wang et al., 2017; Musken et al., 2018). This re-colonization trap is further exacerbated by micro-topographical ‘shadow zones’, microscopic valleys and pits on the implant surface that act as physical sanctuaries, shielding bacterial clusters from the full shear force of interfacial hydrogen evolution (Zhu et al., 2024). Additionally, the *in vivo* environment introduces biological complexities, such as the rapid deposition of host plasma proteins and cellular debris, which can dampen gasification efficiency and shield a critical threshold of viable pathogens from complete electrochemical neutralization. This persistent bacterial presence highlights a critical limitation of autonomous HLES. Although interfacial gasification effectively displaces adherent bacteria and nascent matrices from the implant surface, the detached microorganisms may retain metabolic viability, necessitating adjunctive pharmacological neutralization to preclude secondary colonization. Furthermore, pathogens sequestered within the adjacent bone matrix or soft tissue niches remain shielded from the electrical field’s direct influence, serving as a reservoir for persistent colonization once current delivery ceases (Masters et al., 2022; Yi et al., 2025; Heckmann et al., 2026). This spatial constraint is corroborated by the *in vivo* observations of Weeks et al. (2023), who demonstrated in a rat femoral model that even when low-amperage electrical stimulation is synergized with vancomycin, the prosthesis cleared more readily than the adjacent bone (83% vs. 50% culture negativity). Their findings emphasize that electrical fields and systemic antibiotics clear early surface-adherent bacteria far more effectively than they reach bacteria entrenched in osseous micro-niches.

Similarly, Zhao et al. recently demonstrated in a mouse wound model that high-intensity electrical stimulation (75 mA cm^-2^) alone, despite effectively debriding the wound surface, failed to achieve sustained bacterial reduction, as counts rebounded to baseline levels within 24 hours (Zhao et al., 2023). In contrast, by applying our 1 A pulse and systemic prophylaxis at the point of implantation before mature biofilms or deep osseous infiltration could occur, we achieved total sterilization of both the hardware and the surrounding bone. Collectively, these results suggest that while high-amperage pulses are highly effective for surface decontamination, the total eradication of implant-associated infections requires a multimodal approach capable of addressing both the restored surface and the pathogens sequestered in deep biological niches (Tomizawa et al., 2021; Weeks et al., 2023; Zhao et al., 2023; Zhu et al., 2024).

By evaluating the synergistic potential between high-amperage electrochemical surface clearance and systemic prophylaxis, our work addresses a significant void in the literature and establishes a preemptive framework for circumventing chronic prosthetic colonization. Notably, this combination not only enhances overall eradication rates but also significantly shortens treatment duration, offering a therapeutic strategy that is far more compatible with standard surgical workflows than traditional, prolonged bioelectric protocols.

Finally, although this study focused explicitly on the primary prophylactic window to prevent initial bacterial seeding, the rapid biophysical action of HLES also holds clinical promise for retention protocols. Specifically, in the context of Debridement, Antibiotics, and Implant Retention (DAIR) for acute or early post-operative PJI, HLES, when synergized with targeted antibiotic coverage, could serve as a decisive intraoperative bioelectric adjunct during surgical washout. By leveraging vigorous interfacial gasification to physically strip established biofilm structures from retained hardware, this combined bioelectric-pharmacological approach may overcome the traditional limitations of mechanical debridement and conventional irrigation alone, warranting dedicated investigation in acute infection models.

This study has several limitations, most notably the exploratory nature of this pilot study with one rabbit per treatment condition, which precludes statistical testing, limits adverse event detection, and may not capture inter-individual variability. Importantly, the bacterial inoculum was deliberately refined from 0.5 to 0.3 McFarland between phases. This adjustment was not intended to lower the challenge, but to establish a physiologically representative perioperative contamination level that allowed for the study of early bacterial colonization and initial surface adherence without the confounding effect of the fulminant systemic sepsis observed in early-phase subjects. Additionally, the lack of histological or biomechanical analyses means that microscopic tissue effects or early osseointegration changes cannot be excluded. While these results were obtained with cobalt-chromium implants, outcomes may vary with the use of titanium or other alloy compositions. Finally, follow-up was short, so long-term sterility and integration remain unconfirmed.

Despite these constraints, our model reproducibly generated infection in prosthetic joints and surrounding tissues. Conventional lavage alone could not prevent *S. aureus* colonization; high-amperage current alone produced partial, variable reductions; and systemic antibiotics reduced counts but did not sterilize. Only the integrated approach of lavage, prophylactic antibiotics, and adjunctive high-intensity current achieved complete eradication, preventing both local infection and systemic spread. The observed side-to-side variability underscores the heterogeneity of the surgical micro-environment and supports the need for robust, multimodal prevention strategies. In conclusion, high-amperage pDC represents a potent adjuvant that bridges the efficacy gap left by standard prophylaxis. Refining electrical parameters, expanding pathogen scope, and extending follow-up will be essential steps toward clinical translation of this strategy as a component of perioperative prophylaxis.

## Data Availability

The original contributions presented in the study are included in the article/**Supplementary Material**. Further inquiries can be directed to the corresponding author.
