# Supplementary material for: Adjunctive intraoperative pulsed direct current with cefazolin: a rabbit proof-of-concept model for prevention of prosthetic joint infection
Source: Front Cell Infect Microbiol. 2026 Sep 22;16:1927135. doi: 10.3389/fcimb.2026.1927135 (PMC13638594; doi:10.3389/fcimb.2026.1927135)
Supplement: Supplementary file 1 [file Table1.docx]

Supplementary Material

# 1 Supplementary Tables

Appendix A. Absolute bacterial counts on prosthesis, bone, and soft tissue 72 h after implantation.

| **Group** | **Animal** | **Treatment** | **Knee** | **Prosthesis^a^** | **Bone^b^** | **Soft Tissues^b^** |
| --- | --- | --- | --- | --- | --- | --- |
| No prophylaxis | A1 | Lavage | R | 8.0 × 10^3^ | + | + |
| No prophylaxis | A1 | Lavage | L | 2.9 × 10^5^ | + | + |
| No prophylaxis | A2 | Lavage + pDC | R | 3.3 × 10^1^ | + | + |
| No prophylaxis | A2 | Lavage + pDC | L | 7.7 × 10^5^ | + | + |
| Prophylaxis | B1 | Lavage | R | 2.3 × 10^3^ | 5.0 × 10^2^ | 5.3 × 10^2^ |
| Prophylaxis | B1 | Lavage | L | 1.5 × 10^3^ | 1.8 × 10^3^ | 7.4 × 10^3^ |
| Prophylaxis | B2 | Lavage + pDC | R | 0 | 0 | 0 |
| Prophylaxis | B2 | Lavage + pDC | L | 0 | 0 | 0 |

^a^CFU cm^-2^; ^b^CFU mL^-1^. pDC: pulse direct current. R: Right; L: Left.

Appendix B. Quantitative bacterial burden 72 h after implantation, expressed log_10_(CFU cm^-2^) for prostheses and log_10_(CFU mL^-1^) for bone and soft tissue.

| **Group** | **Animal** | **Treatment** | **Knee** | **Prosthesis^a^** | **Bone^b^** | **Soft Tissues^b^** |
| --- | --- | --- | --- | --- | --- | --- |
| No prophylaxis | A1 | Lavage | R | 3.90 | + | + |
| No prophylaxis | A1 | Lavage | L | 5.46 | + | + |
| No prophylaxis | A2 | Lavage + pDC | R | 1.52 | + | + |
| No prophylaxis | A2 | Lavage + pDC | L | 5.89 | + | + |
| Prophylaxis | B1 | Lavage | R | 3.36 | 2.70 | 2.72 |
| Prophylaxis | B1 | Lavage | L | 3.20 | 3.26 | 3.87 |
| Prophylaxis | B2 | Lavage + pDC | R | ND | ND | ND |
| Prophylaxis | B2 | Lavage + pDC | L | ND | ND | ND |

^a^Log_10_ (CFU cm^-2^); ^b^Log_10_ (CFU mL^-1^). pDC: pulse direct current. R: Right; L: Left. ND: not detected.
